# The evolution of parental care in salamanders

**DOI:** 10.1038/s41598-022-20903-3

**Published:** 2022-10-05

**Authors:** Balázs Vági, Daniel Marsh, Gergely Katona, Zsolt Végvári, Robert P. Freckleton, András Liker, Tamás Székely

**Affiliations:** 1 ELKH-DE Reproductive Strategies Research Group, Department of Evolutionary Zoology and Human Biology, University of Debrecen, 4032 Debrecen, Hungary; 2 Milner Centre for Evolution, University of Bath, Bath, BA2 7AY UK; 3 Centre for Ecological Research, Institute of Aquatic Ecology, Karolina út 29, 1113 Budapest, Hungary; 4 Senckenberg Deutsches Entomologisches Institut, 15374 Müncheberg, Germany; 5 School of Biosciences, University of Sheffield, Sheffield, S10 2TN UK; 6 ELKH-PE Evolutionary Ecology Research Group, University of Pannonia, Pf. 1158, 8210 Veszprém, Hungary; 7 Behavioural Ecology Research Group, Center for Natural Sciences, University of Pannonia, Pf. 1158, 8210 Veszprém, Hungary

**Keywords:** Evolution, Ecology, Evolutionary ecology

## Abstract

Complex parenting has been proposed to contribute to the evolutionary success of vertebrates. However, the evolutionary routes to complex parenting and the role of parenting in vertebrate diversity are still contentious. Although basal vertebrates provide clues to complex reproduction, these are often understudied. Using 181 species that represent all major lineages of an early vertebrate group, the salamanders and newts (Caudata, salamanders henceforth) here we show that fertilisation mode is tied to parental care: male-only care occurs in external fertilisers, whereas female-only care exclusively occurs in internal fertilisers. Importantly, internal fertilisation opens the way to terrestrial reproduction, because fertilised females are able to deposit their eggs on land, and with maternal care provision, the eggs could potentially develop outside the aquatic environment. Taken together, our results of a semi-aquatic early vertebrate group propose that the diversity and follow-up radiation of terrestrial vertebrates are inherently associated with a complex social behaviour, parenting.

## Introduction

Parental care is one of the most diverse social behaviours^1–3^. The various forms of care evolved to protect, nurture and educate offspring which led to the development of parental strategies including diverse morphological, physiological and behavioural adaptations to increase offspring development and survival^4–7^. Parental care is ubiquitous in endotherm vertebrates (i.e., birds and mammals), while it is far less frequent in ectotherms including fishes, amphibians and invertebrates^8–14^. Due to the diversity of parental care, including the extent and duration of parenting of early vertebrates that include fishes and amphibians, these aquatic and semi-aquatic groups represent ideal model systems for studying the origins and diversification of parental care as well as the social and environmental predictors of parenting^11,15^.

Parental care often emerges to cope with abiotic environments such as harsh and/or unpredictable environments that are characterised by extremities, or extensive fluctuations in temperature and/or water availability^16,17^. In order to reproduce in non-aquatic environments and thus invade terrestrial niches, early vertebrates faced substantial challenges because anamniote eggs laid in terrestrial environments would quickly desiccate, be subject to stalled embryonic development due to extreme ambient temperatures, or become infected by microbes and consumed by various predators^18,19^. Forms of parental care has evolved to overcome these challenges and protect the eggs from such threats, e.g., brooding of the clutch by physical contact or urination, removing infected eggs, or active defence of the clutch from predators^20,21^. Changing the breeding environment from aquatic to terrestrial egg-laying was a key transition by early tetrapods toward colonising land-based ecological niches, and it was often accompanied by changes in basic life history traits such as reproductive outputs including egg and clutch size^11,22^. Consequently, contemporary groups of basal vertebrates that exhibit both aquatic and non-aquatic reproduction could provide excellent model systems for understanding the evolutionary drivers of these transitions and the role of parental care in the process.

Here we focus on newts and salamanders (Caudata or Urodela, henceforth salamanders) that are a sister group of frogs and toads (Anura). The approx. 750 species of salamanders live in the Palearctic, Nearctic and (with less diversity) in the Neotropical realms, and they exhibit diverse reproductive strategies that include both internal and external fertilisations, viviparity, and a variety of developmental modes and parenting types^23^. Our overall objective here is to explore the life history, climatic and reproductive correlates of parenting in salamanders. Whilst previous studies of salamander reproduction were insightful^10,23–27^, they left two main questions unresolved. First, we still do not know if parental care is associated with reproductive modes, in particular with the mode of fertilisation and various forms of offspring development. Second, climatic and life history traits have been hypothesised to predict parental care variation^11,12,23–26^, although these associations have not been investigated quantitatively using a broad range of salamander taxa in an explicit phylogenetic framework.

Here we have three objectives. First, to investigate whether parental care by the male and/or the female relates to fertilisation mode (external/internal). Although previous studies have uncovered associations between fertilisation and male versus female care in fishes and frogs^9,28^, salamanders provide an independent taxon to assess these relationships. Specifically, we hypothesised that external fertilisation is associated with male care, whereas internal fertilisation with female care^9,29^. Second, parental care is often associated with life history traits, e.g. larger reproductive investment into individual offspring by providing more nutrients that supply the developing embryo as indicated by large eggs^13,30^. Thus, we predicted that parental care is associated with large eggs and with small clutches owing to the trade-off between egg size and clutch size^13,22,27,30^. Finally, we also investigate the role of climate: based on previous studies, we predicted that harsh environment as indicated by less precipitation and/or uneven distribution of precipitation is associated with extensive parenting^16,17,31,32^. Therefore, we predicted that parenting is associated with terrestrial reproduction and occurs in dry and seasonally fluctuating ambient environments^11,12,18,19^.

## Materials and methods

### Data collection

We collected data on salamander parental care from the available literature, including books, primary papers and refereed online sources (see electronic supplementary material). We consider that our data has a representative phylogenetic coverage, as the 181 species in our dataset (out of approximately 750 species) represent all salamander families and all major lineages within them.

We defined parental care as the attendance of the eggs, because—with the exception of the two viviparous clades—this is the only widespread care type known in salamanders, as parents usually do not provide care after the hatching of the eggs. Therefore, we classified care as: no care, male-only care and female-only care. The two known viviparous lineages of salamanders (*Salamandra* + *Lyciasalamandra* in Salamandridae, and *Bolitoglossa peruviana* in Plethodontidae) were excluded from the analyses, because egg attendance is not relevant in their cases. Note that the inclusion of these species does not qualitatively change the main conclusion of this study, as all viviparous species have internal fertilisation and terrestrial reproduction.

Juvenile attendance may occur in extremely rare cases^33^, however, as it is only a short extension of egg attendance, we did not denote it as a separate type of care. We considered attendance as absent where this is explicitly stated in the sources or when it is not referred to in otherwise detailed descriptions of the reproductive behaviour that included observations on courtship and data on clutch size and egg-laying substrate.

In transition rate analyses and character reconstructions, we classified parental care as a three-level trait (no care, male care, female care). In some species we found conflicting information on the caregiver sex. We classified these species according to newer, clearly confirmed information^34^, or, when we could not make a univocal decision, we excluded them from the analyses (*Proteus anguinus*, *Bolitoglossa subpalmata*^10,35^ and references therein). In one species (*Bolitoglossa pesrubra*^36^, cited by^35^) plasticity is reported in the caregiver sex, however, in 87% of the observations care is provided by females, thus, we classified this species as having female care.

We classified fertilisation as external or internal (the latter only indicates the transmission of a spermatophore in salamanders^24^). We collected clutch size and egg-diameter data from the literature. Egg diameter was measured as the size of the vitelline (in mm). When we found a range given for egg and clutch size, we calculated mean values. Clutch volume was measured as egg volume (calculated from egg diameter by assuming a spherical shape) multiplied by clutch size. As egg size and clutch volume is strongly dependent on body size in amphibians^24,37–39^, when investigating the effect of relative reproductive output, we used a body size metric as a control variable. We defined cubed male or female snout-to-vent length as a proxy for body volume in analyses for egg or clutch volume. We also used sex-specific snout-to-vent length (SVL) for calculating sexual size dimorphism, which is often associated with the breeding system. Sexual size dimorphism was calculated as log_10_(male SVL / female SVL)^40,41^.

We collected information on offspring developmental mode from the literature (see electronic supplementary material). We coded developmental mode in two alternative ways using ordinal and binary variables. For the 3-level ordinal coding, all egg-laying species were classified as having 1–aquatic (aquatic eggs and larvae), 2–semiterrestrial (terrestrial eggs and aquatic larvae) or 3–terrestrial (direct development from terrestrial eggs to terrestrial juveniles) development. In the analyses of evolutionary transition rates (see below), we used a binary variable instead, aquatic or terrestrial reproduction (based on the egg-laying site; thus, we merged categories 2 and 3).

We used annual mean temperature and annual sum of precipitation, their within-year variances and between-year variances calculated from monthly values^42^ as potential predictors of parental care variation, given these variables proved to be important predictors of the developmental mode and care providing in frogs^12,22,43^. Spatial climatic data was processed using the R packages ‘maptools’, ‘raster’ and ‘rgdal’^44–46^. We obtained geographic ranges for 174 salamander species at iucnredlist.org^47^. We excluded range polygons that include introduced and unconfirmed populations, to exclude evolutionary irrelevant or unconfirmed records^12^. We downloaded global temperature and precipitation data from WorldClim^48^ in 2.5’ × 2.5’ resolution rasters, which we cropped to the species’ adjusted distribution ranges. We calculated annual mean temperatures, precipitation sums, within-year variance of monthly values and between-years variance of annual values across the past 50 years for each raster cells, and then calculated average values for each species’ distribution rasters^12^. Because our calculation produced unreliable data (i.e. 0 or close to 0 mm annual precipitation sums) for species with very small ranges, we excluded species with ranges smaller than 0.1 km^2^ (N = 34 species) from the climatic analyses.

### Phylogenetic analyses

To account for the phylogenetic non-independence, in ancestral state reconstructions we used the comprehensive consensus tree of Jetz and Pyron^49^ which contains 94% of known amphibian species. We calculated transition rates between combinations of character states and ancestral trait value combinations using the R package ‘CorHMM’^50^. It uses hidden Markov models (HMMs) to reconstruct evolutionary transitions between discrete character states and excludes the simultaneous transitions in two traits. We investigated character combinations between parental care types and (a) fertilisation modes; (b) terrestrial egg laying. In (b) we applied a binary coding for terrestrial egg laying (see above).

To test associations between parental care forms and (i) breeding systems: fertilisation and sexual size dimorphism; (ii) life history; (iii) climatic environment and (iv) the combination of multiple factors, we used phylogenetically informed analyses which control for the phylogenetic dependence among the species and their traits. Because our response variables were based on the presence or absence of male and female care, we used phylogenetic generalized linear (PhyloGLM) models in the R package “phylolm”^51^ which can handle binary response variables. In these models, we entered the binary-coded “male parental care” or “female parental care”, respectively, as the response variable. For these analyses, we generated a sample of 100 phylogenetic trees from VertLife.org^52^ to assess phylogenetic variance within a given model design.

As our data coverage was better for reproductive modes than for life history and climate, first we analysed the effects of (i) reproductive mode (fertilisation and offspring development); (ii) life history (body size, egg size, clutch size and clutch volume; sexual size dimorphism) and (iii) the climate in separate models to maximise species numbers and increase power of the tests. We constructed 7 + 7 model designs for male and for female care (Table 1). In the first set (models 1–2, including models for both males and females), we investigated characteristics of reproductive modes, namely fertilisation and offspring development (coded by the 3-level ordinal variable). We also investigated the effect of fertilisation and offspring development applying bivariate models (models S1-S4 in Supplementary Table S1). In the next set of models, we investigated variables describing life-history traits. In models 3–10, we used body volume, egg volume, clutch size or clutch volume as predictors. In models with egg and clutch volume we included male or female body volume (in the models on male and female care, respectively), to control for the total body size, which strongly influences both egg size and the total reproductive output. However, we did not include a body size variable in the models with clutch size, because we aimed to investigate the effect of total egg (and offspring) numbers. We also investigated the effect of sexual size dimorphism on male and female parental care (models 11–12). In two further multipredictor models we combined fertilisation and multiple variables describing life history (models S5–S6 in Supplementary Table S1). We used one measure for reproductive output: egg size (measured as egg volume) in each of these two multipredictor models. Note that these models had limited species numbers compared to models 1–2 and S1–S4.

**Table 1:** Predictors of female and male care in salamanders.

Response variable	Male care			Female care		
Predictors	*β* ± *SE*	*Phylogenetic variance*	*p*	*β* ± *SE*	*Phylogenetic variance*	*p*
**Reproductive mode**	Model 1 (N = 180)			Model 2 (N = 180)		
Fertilisation	**− 4.120 ± 1.908**	**1.102**	**0.032**	**3.734 ± 1.782**	**0.095**	**0.038**
Offspring development	0.025 ± 1.149	0.284	0.983	**0.716 ± 0.328**	**0.041**	**0.030**
**Life history**	Model 3 (N = 132)			Model 4 (N = 135)		
Body size	**1.741 ± 0.650**	**0.008**	**0.008**	0.052 ± 0.248	0.113	0.836
	Model 5 (N = 118)			Model 6 (N = 121)		
Egg size	− 0.799 ± 1.023	0.140	0.437	0.075 ± 0.382	0.223	0.845
Body size	**2.017 ± 0.799**	**0.071**	**0.013**	− 0.072 ± 0.474	0.390	0.879
	Model 7 (N = 162)			Model 8 (N = 162)		
Clutch size	0.541 ± 0.860	0.417	0.530	− 0.000 ± 0.240	0.001	0.999
	Model 9 (N = 109)			Model 10 (N = 112)		
Clutch volume	− 0.589 ± 0.432	0.057	0.176	− 0.134 ± 0.324	0.047	0.680
Body size	*0.810* ± *0.463*	*0.044*	*0.083*	0.163 ± 0.315	0.058	0.607
	Model 11 (N = 132)			Model 12 (N = 132)		
Sexual size dimorphism	− 0.001 ± 0.547	0.004	0.999	− 0.372 ± 0.983	0.143	0.706
**Climate**	Model 13 (N = 111)			Model 14 (N = 111)		
Annual T_mean_	0.112 ± 0.119	0.009	0.349	**− 0.174 ± 0.086**	**0.000**	**0.046**
Within-year T_mean_ variance	0.583 ± 0.499	0.001	0.245	0.181 ± 0.299	0.000	0.546
T_mean_ stochasticity	0.504 ± 0.491	0.004	0.307	0.028 ± 0.336	0.000	0.933
Annual precipitation	0.004 ± 0.003	0.000	0.138	**0.003 ± 0.002**	**0.000**	**0.036**
Prec_ann_ stochasticity	0.046 ± 0.030	0.000	0.135	− 0.015 ± 0.022	0.000	0.485
Terrestrial eggs				4.823 ± 0.956	0.000	< 0.001

Phylogenetic generalized linear models (phyloGLM) of female-only or male-only care (response variables) in relation to reproductive modes (models 1–2), life history (models 5–12) and climatic environment (models 13–14) with a set of 100 phylogenetic trees. See additional models in Supplementary Table S1.

We provide number of species (N), parameter estimates with standard error (*β* ± *SE*), phylogenetic variance and *p* values. Bold fonts represent significant (*p* < 0.05), *cursive* fonts represent marginally significant (*p* < 0.1) statistics. Egg size and body size were estimated as volumes (see Materials and methods). In models of male care, we used male body volume, while in models on female care we used female body volume.

Finally, we built multipredictor models with climatic variables as the predictors (models 13–14). We excluded within-year precipitation variance from these models due to its strong correlation with total annual precipitations sums (r > 0.7). Since species with female care can have either aquatic or terrestrial egg-laying, and the effect of climatic factors may be different in these two types of environment, we also included terrestrial egg-laying as a factor in the analyses on female parental care (but note that we present models without this factor in the Supplementary Information). Because climatic variables often have complex effects, we also performed a model selection on the full set of candidate models with all combinations of climatic variables. Model performance was ranked based on AICc values and models with 2 > ΔAICc values were considered to have support.

All analyses were carried out using the R 4.0.4 statistical programming environment^53^.

## Results

**Figure 1:**
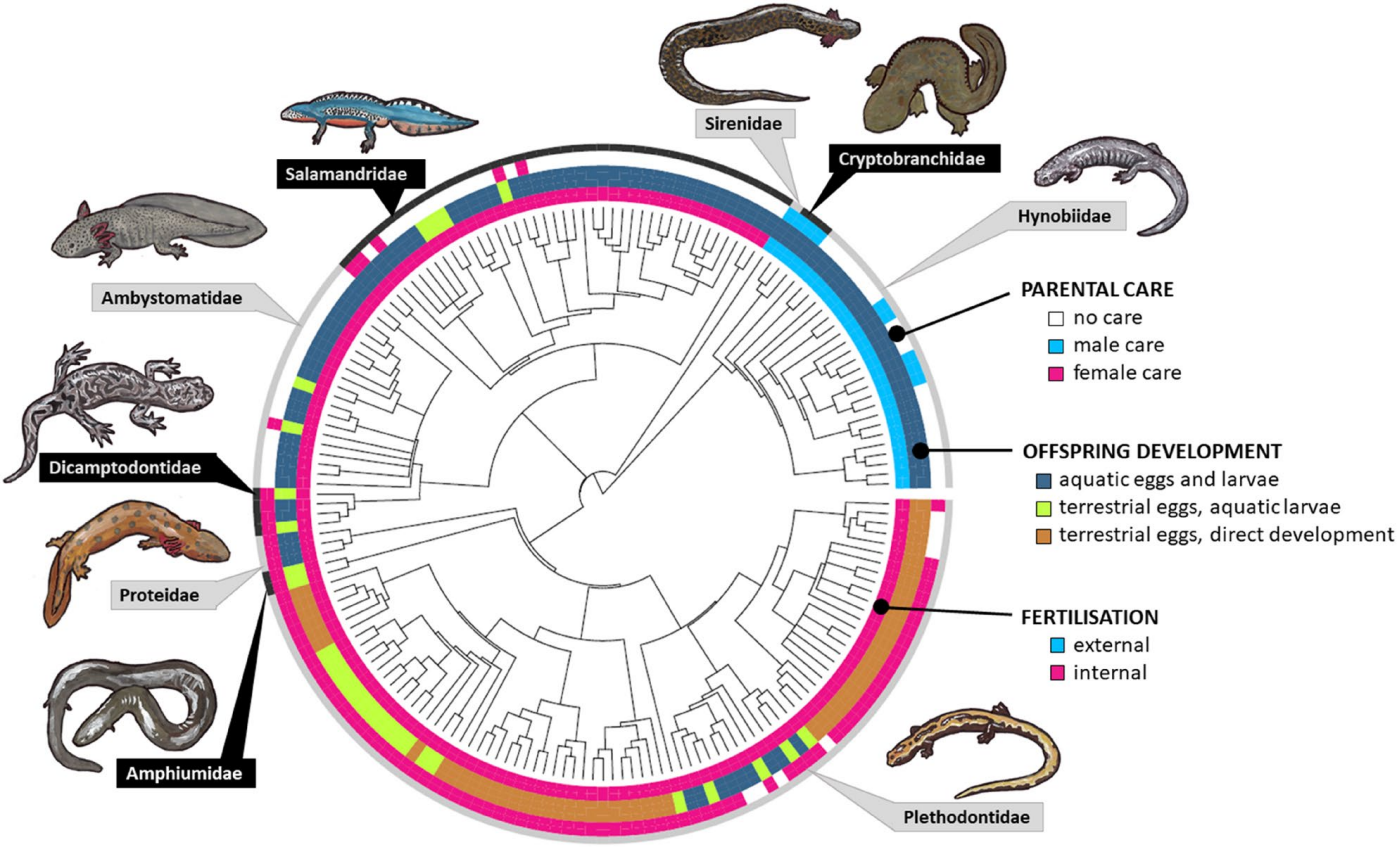
The distribution of care, offspring development and fertilisation in 179 species of salamanders and newts (Caudata) that represent 9 families. Phylogenetic tree was created in the R 4.0.4 statistical programming environment (https://cran.r-project.org/) using the package ‘diversitree’. Original artwork was made by Balázs Vági.

**Figure 2:**
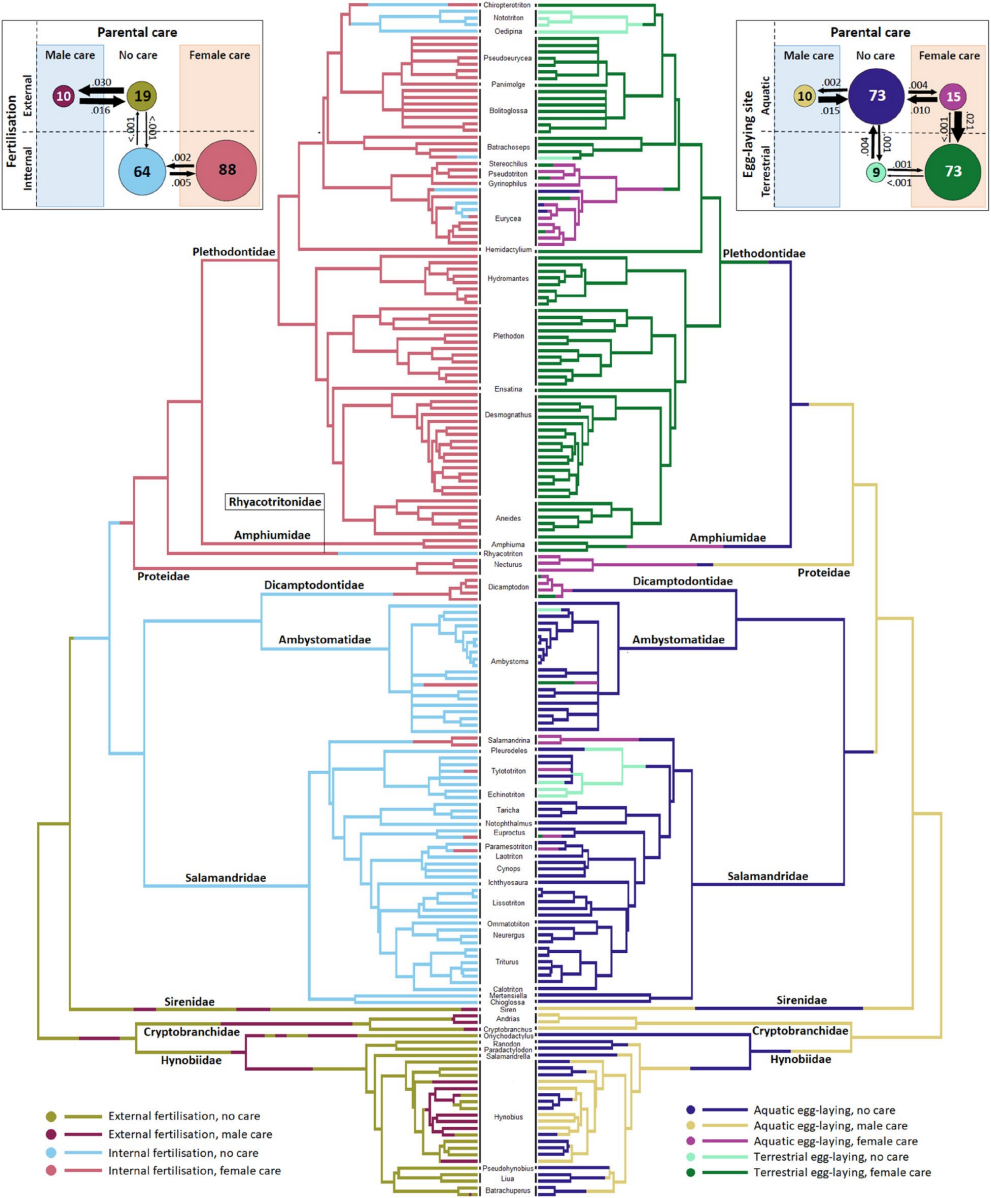
Character state reconstruction for trait combinations of parental care and fertilisation (left panel), and reproduction (right panel) in 181 species of salamanders and newts. Inset figures: evolutionary transition rates inferred between care and fertilisation (left), and care and egg-laying site. Branches of the tree and the circles of the inset figure refer to the same colour coding, while the size of the circles and numbers within them represent the number of contemporary species. Numbers above the arrows represent evolutionary transition rates. Original figure was created in the R 4.0.4 statistical programming environment (https://cran.r-project.org/) using the package ‘corHMM’ and ‘ape’.

Out of 181 species of salamanders 46% (83 species) exhibit no care, whilst 5.5% and 48.5% (10 and 88 species, respectively) exhibit male care and female care (Fig. 1). We are not aware of any biparental salamander species. Character state reconstructions suggest that either male attendance or no care could have been the ancestral state in the common ancestor of salamanders (Fig. 2), whereas male and female care evolved multiple times from no care (Fig. 2). The estimated evolutionary transition rates indicate that fertilisation mode is rather rigid in evolutionary terms, while egg-laying site and parental attendance are more flexible in evolutionary terms (Fig. 2). Note that no direct transitions between male care and female care have been inferred (Fig. 2).

Parental care is tightly associated with fertilisation mode because male care occurs exclusively in ancient families with external fertilisation (Hynobiidae, Cryptobranchidae and Sirenidae, whereas female care only occurs in clades with internal fertilisation (Figs. 1, 2). We recorded maternal attendance of the eggs in most families with internal fertilisation: Salamandridae, Ambystomatidae, Dicamptodontidae, Proteidae, Amphiumidae, and Plethodontidae. Among the more speciose families, maternal care rarely occurs in the Salamandridae and Ambystomatidae, but it is provided by the vast majority of Plethodontidae (Figs. 1, 2).

Parental care is also associated with offspring development because male care is associated with aquatic egg-laying; whereas female care occurs with either aquatic or terrestrial eggs (Fig. 2). Importantly, female care facilitated the transitions from aquatic egg-laying to terrestrial egg laying (Fig. 2). The results of phylogenetic models on fertilisation mode and offspring development support the hypothesis that fertilisation predicts care type: external fertilisation predicts male care, whereas internal fertilisation predicts female care (Table 1: models 1–2 and Supplementary Table S1: models S1–S4).

The only significant life history predictor of care is male size: species with large males are more likely to provide than species with small males (Table 1: models 3–12). In multipredictor models containing both reproductive modes and life history variables the effects of any predictor are no longer significant (Supplementary Table S1: models S5–S6); however, note that decreased species numbers due to the gaps in life history data reduce the power of these tests.

Finally, according to climatic models, none of the climatic variables predict male care, while female care is associated with the climate, however, terrestrial reproduction mediates this association (Table 1: models 13–14; Supplementary Tables S1–S3). In climatic models which also include terrestrial egg-laying as a predictor, female attendance is predicted by lower annual temperatures and higher precipitation sums (Table 1: model 14; Supplementary Tables S3). The model selection process confirmed the importance of low annual mean temperatures as a predictor of female parental care (Supplementary Table S5).

## Discussion

### Parental care, fertilisation and offspring development

Our results show that externally fertilising salamanders did not switch to terrestrial reproduction so that terrestrial egg-laying was only achieved after the fertilisation mode shifted to internal. However, after the transition in fertilisation mode, it was parental care that facilitated independence from aquatic habitats; in a similar way to other basal vertebrates^11,19,22^. While parental care may have benefits in an aquatic environment, as oxygenation or protection from predation and infections^8,9,54,55^, it seems an even more important innovation for reproduction outside the aquatic environment which is hostile for anamniotic eggs^18,20,21^. According to observations in some attending salamander species, protection of terrestrial clutches by active defence against predators or by the removal of mould-infected eggs indeed increase hatching success^56–58^. Nonetheless, we could not rule out an opposite causality, whereby male or female parental care select for external or internal fertilisation, respectively. However, the latter scenario seems unlikely based on the inferred direction and transition rates between fertilisation and care. In addition, parental care appears to be more flexible on an evolutionary timescale than fertilisation mode.

In salamanders, uniparental male and female care were inferred to evolve from no care multiple times without direct transitions between the two types. This observation is in line with the central role of fertilisation mode in determining parental roles—as fertilisation mode proved to be rigid in evolutionary terms, it also hampered transitions in the care-providing sex. The emergence of male care in ectotherms has been argued to be explained by certainty of paternity, which is in turn linked to breeding systems. For instance, in bony fishes, male care is associated with pair spawning^9^. In addition, males can attend egg clutches from multiple females, maximising their reproductive success by polygyny^15^. While guarding behaviour itself can increase the male’s attractiveness as a good quality parent in other groups^59^, its potential importance in female choice has not been tested in salamanders, although male care also seems to have coevolved with polygyny in some urodelans^60,61^. As in external fertilizers, the eggs are exposed to sneaker males and clutch piracy^62,63^, ensuring high paternity could have been an important driving force of the evolution of male egg attendance. By defending the clutch from other males and also from predators, large salamander males can be more efficient in parenting than small males, similar to fish^64,65^. Nonetheless, in salamanders the latter transitions were not associated with increased sexual size dimorphism^11,37^—one trait that is often (but not always) associated with intense sexual selection^66^.

Biparental care has never evolved in salamanders. Although the caregiver sex shows some plasticity in a small number of species^36^ so that one parent can fully take over the other’s duties, nevertheless the care remains uniparental (amphisexual care^67–69^). Stable pair bonds and social monogamy are unknown in salamanders; these social structures were found to be associated with biparental care in other taxa^64,70,71^. Moreover, in many biparental taxa, the parents provide complementary care functions^10,13^, and one of these functions is usually the feeding of the offspring. In contrast to frogs and caecilians, offspring feeding has never evolved in salamanders, with the possible exception of matrotrophy, which does occur in viviparous salamanders^72^. While most frog tadpoles feed on algae and detritus and easily adapt to food sources provided by the parents by secretions^73,74^ or by trophic eggs^75,76^, all free-living salamander larvae and juveniles are predators of small, moving prey^24–26^. Possibly the predatory habit of salamander larvae contributed to the lack of parental feeding in this group. Future studies of amphibian larvae development that investigates larvae feeding habits in urodelans and anurans are warranted; and also in caecilians in which dermatophagy (offspring feeding on maternal skin) occurs in a number of species^39,77^.

### Parental care, life history and climate

Interestingly, we did not uncover associations between egg size, clutch size and parental care. Although associations between large eggs and parental care are assumed to be widespread among ectotherms^14,27,30^, they may be mediated by a common underlying factor, such as terrestrial reproduction^11^. In salamanders, the association between large eggs and terrestrial egg laying may not be straightforward since stream-type aquatic environments also select for large eggs^24–26^. Additionally, although larger eggs and smaller clutches are hypothesised to have coevolved with any form of parental care^78^, more recent research indicate that these seem more important predictors of parental care in species which also provide nourishment than in the ones that merely guard their clutch^11,13^. Therefore, the absence of offspring nourishment may also explain the general lack of association between parental care and reproductive output in salamanders.

Our climatic models suggest that female care, which mostly occurs in terrestrial environments among salamanders, is more climate dependent then male care, which is always aquatic in this group. Female attendance occurs in cooler and wetter habitats, thus, it seems that active protection could not be provided in very hostile environments as the parent is also vulnerable to overheating and desiccation. Rather, when the climate is favourable, the attending parent can ensure higher survival by protecting the clutch from other threats such as predation and infections, like in frogs^12^. Note however, that the climate data we used is of relatively raw resolution (approx. 2.3 km grid size), and the dataset we used may only give a crude estimate of the climatic conditions relevant for each species. In addition, it is also possible that the social environment, i.e., mating opportunities, have also shaped the evolution of male and female parental care, as has been found in other early vertebrate groups^9,12,79–81^. Unfortunately, information on genetic and social mating systems, or other details of life history such as longevity or pace-of-life are largely missing for most salamander species. Future analyses will be needed to use population-level climatic data—preferably extracted for the same population that provides the data on reproduction, life histories and parenting.

Taken together, we hope that our analyses will shed light on the selective forces that may operate in salamanders, that have somewhat secretive lifestyles. Although many new species have been described recently both in diverse and species-poor clades^82–84^, their diversity is alarmingly threatened, while their natural histories often remain unexplored^83,85^.

## Conclusions

In summary, our results confirm the central role of fertilisation in driving the evolution of parental care. In addition, we identified sex-specific predictors of male and female parental care. Thus it seems that internal fertilisation and female egg attendance were the two key transitions for a terrestrial life style in salamanders, facilitating radiations towards a variety of niches^84,86^. We call for future studies of various vertebrate groups to uncover the drivers of their diversification and evolutionary success.

## Supplementary Information

Supplementary Information 1.

Supplementary Information 2.

## Data Availability

All the analysed data are available from the corresponding author and will be placed to a public repository after the acceptance of the manuscript.
