# Supplementary material for: The evolution of parental care in salamanders
Source: Sci Rep. 2022 Oct 5;12:16655. doi: 10.1038/s41598-022-20903-3 (PMC9535019; doi:10.1038/s41598-022-20903-3)
Supplement: Supplementary file 2 — Supplementary Information 2. [file 41598_2022_20903_MOESM2_ESM.docx]

**Vági et al. The evolution of parental care in salamanders**

**Supplementary Information**

**Supplementary Table S1.** Predictors of female care and male care in salamanders. Phylogenetic generalized linear models (phyloGLM) of female or male parental care (response variables) in relation to reproductive modes (models S1–S4), reproductive modes and life history combined (models S5–S6) and climatic environment (models S7–S16) with a set of 100 phylogenetic trees.

| Response variable: | **Male care** | | | | | | |  | **Female care** | | | | |
| --- | --- | --- | --- | --- | --- | --- | --- | --- | --- | --- | --- | --- | --- |
| Predictors: |  | *β ± SE* | *phylogenetic.variance* | | *p* | |  | | *β ± SE* | | *phylogenetic.variance* | | *p* |
| **Reproductive mode** |  | Model S1 (N = 181) | | | | |  |  | Model S2 (N = 181) | | | | |
| Fertilisation |  | **-5.067 ± 1.551** | **0.209** | | **0.001** | |  | | *3.710 ± 1.934* | | *0.241* | | *0.057* |
|  |  | Model S3 (N = 180) | | | | |  | | Model S4 (N = 180) | | | | |
| Offspring development |  | -0.001 ± 0.547 | 0.004 | | 0.999 | |  | | **0.575 ± 0.276** | | **0.022** | | **0.038** |
| **Multipredictor models** |  | Model S5 (N = 117) | | | | |  | | Model S6 (N = 120) | | | | |
| Internal fertilisation |  | -1.950 ± 1.429 | 0.358 | | 0.173 | |  | | 2.539 ± 2.116 | | 1.436 | | 0.234 |
| Offspring development |  | 0.291 ± 0.619 | 0.448 | | 0.639 | |  | | 0.432 ± 0.591 | | 0.473 | | 0.466 |
| Egg size |  | -0.657 ± 0.390 | 0.076 | | 0.095 | |  | | 0.252 ± 0.481 | | 0.269 | | 0.601 |
| Body size |  | 0.658 ± 1.413 | 0.757 | | 0.510 | |  | | 0.148 ± 0.478 | | 0.317 | | 0.758 |
| **Climate** |  | Model S7 (N = 111) | | | |  | | | Model S8 (N = 111) | | | | |
| Annual T_mean_ |  | 0.000 ± 0.082 | 0.002 | 1.000 | |  | | | -0.010 ± 0.028 | 0.009 | | 0.710 | |
|  |  | Model S9 (N = 111) | | | |  | | | Model S10 (N = 111) | | | | |
| Within-year T_mean_ variance |  | 0.001 ± 0.241 | 0.004 | 0.996 | |  | | | 0.000 ± 0.081 | 0.001 | | 0.998 | |
|  |  | Model S11 (N = 111) | | | |  | | | Model S12 (N = 111) | | | | |
| T_mean_ stochasticity |  | -0.001 ± 0.213 | 0.004 | 0.995 | |  | | | -0.004 ± 0.091 | 0.011 | | 0.965 | |
|  |  | Model S13 (N = 111) | | | |  | | | Model S14 (N = 111) | | | | |
| Annual precipitation |  | 0.002 ± 0.003 | 0.000 | 0.452 | |  | | | 0.001 ± 0.001 | 0.000 | | 0.487 | |
|  |  | Model S15 (N = 111) | | | |  | | | Model S16 (N = 111) | | | | |
| Prec_ann_ stochasticity |  | 0.040 ± 0.032 | 0.000 | 0.210 | |  | | | -0.005 ± 0.010 | 0.000 | | 0.994 | |

We provide number of species (N), parameter estimates with standard error (*β ± SE),* phylogenetic variance and *p* values. **Bold** fonts represent significant (*p* < 0.05), *cursive* fonts represent marginally significant (*p* < 0.1) statistics. Egg size and body size were estimated as volumes (see Materials and methods). In models of male care, we used male body volume, while in models on female care we used female body volume.

**Supplementary Table S2.** Multipredictor model on female care in salamanders including climatic predictors and without the effect of aquatic or terrestrial egg-laying. Phylogenetic generalized linear model (phyloGLM) of female parental care (response variables) in relation to climatic factors with a set of 100 phylogenetic trees.

| Response variable: | **Female care** | | | |  |
| --- | --- | --- | --- | --- | --- |
| Predictors: |  | *β ± SE* | *phylogenetic.variance* | *p* | |
| **Climate** |  | Model S16 (N = 111) | | | |
| Annual T_mean_ |  | **-0.101 ± 0.048** | **0.000** | **0.038** | |
| Within-year T_mean_ variance |  | **-0.391 ± 0.183** | **0.000** | **0.035** | |
| T_mean_ stochasticity |  | -0.277 ± 0.193 | 0.000 | 0.153 | |
| Annual precipitation |  | 0.001 ± 0.001 | 0.001 | 0.136 | |
| Prec_ann_ stochasticity |  | -0.008 ± 0.010 | 0.000 | 0.429 | |

We provide number of species (N), parameter estimates with standard error (*β ± SE),* phylogenetic variance and *p* values. **Bold** fonts represent significant (*p* < 0.05), *cursive* fonts represent marginally significant (*p* < 0.1) statistics

**Supplementary Table S3.** Models on individual climatic predictors of female care in salamanders including the effect of aquatic or terrestrial egg-laying. Phylogenetic generalized linear models (phyloGLM) of female parental care (response variables) in relation to climatic factors and egg-laying site combined with a set of 100 phylogenetic trees.

| Response variable: | **Female care** | | |
| --- | --- | --- | --- |
| Predictors: | *β ± SE* | *phylogenetic.variance* | *p* |
|  | Model S18 (N = 110) | | |
| Annual T_mean_ | **-0.105 ± 0.046** | **0.020** | **0.022** |
| Terrestrial eggs | **1.523 ± 0.630** | **0.317** | **0.017** |
|  | Model S19 (N = 110) | | |
| Within-year T_mean_ variance | 0.166 ± 0.136 | 0.046 | 0.223 |
| Terrestrial eggs | 0.935 ± 0.676 | 0.440 | 0.170 |
|  | Model S20 (N = 110) | | |
| T_mean_ stochasticity | 0.258 ± 0.165 | 0.064 | 0.120 |
| Terrestrial eggs | **1.386 ± 0.648** | **0.316** | **0.035** |
|  | Model S21 (N = 110) | | |
| Annual precipitation | *0.003 ± 0.001* | *0.000* | *0.062* |
| Terrestrial eggs | **4.085 ± 0.808** | **0.000** | **< 0.001** |
|  | Model S22 (N = 110) | | |
| Prec_ann_ stochasticity | **-0.027 ± 0.013** | **0.006** | **0.038** |
| Terrestrial eggs | **2.381 ± 0.939** | **0.653** | **0.013** |

We provide number of species (N), parameter estimates with standard error (*β ± SE),* phylogenetic variance and *p* values. **Bold** fonts represent significant (*p* < 0.05), *cursive* fonts represent marginally significant (*p* < 0.1) statistics

**Supplementary Table S4.** Climatic models on male care in salamanders ranked by AICc values. Supported models (within *Δi* < 2 from the best performing model) are highlighted with **bold** fonts. Abbreviations for climatic variables: *tmean.ann*: annual mean temperature; *tvar.ann*: within-year variance of monthly temperature means; *tmn.st.ann*: between-year stochasticity of annual mean temperatures; *prec.ann*: annual precipitations sums; *prec.st.ann*: between-year stochasticity of annual precipitation sums.

| *Rank* | *Model formula* | *AICc* | *Δi* |
| --- | --- | --- | --- |
| **1** | **Male care ~ prec.st.ann** | **33,13** | **0,00** |
| **2** | **Male care ~ tvar.ann** | **33,20** | **0,07** |
| **3** | **Male care ~ tmean.ann** | **33,28** | **0,15** |
| **4** | **Male care ~ tmn.st.ann** | **34,22** | **1,09** |
| **5** | **Male care ~ tmean.ann+prec.st.ann** | **34,50** | **1,37** |
| **6** | **Male care ~ tmn.st.ann+prec.st.ann** | **34,71** | **1,59** |
| **7** | **Male care ~ tvar.ann+tmn.st.ann** | **35,02** | **1,90** |
| 8 | Male care ~ tvar.ann+prec.st.ann | 35,19 | 2,06 |
| 9 | Male care ~ prec.ann | 35,29 | 2,16 |
| 10 | Male care ~ tmean.ann+tmn.st.ann | 35,34 | 2,21 |
| 11 | Male care ~ tmean.ann+tvar.ann | 35,34 | 2,21 |
| 12 | Male care ~ tvar.ann+tmn.st.ann+prec.st.ann | 35,74 | 2,61 |
| 13 | Male care ~ tmn.st.ann+prec.ann+prec.st.ann | 35,80 | 2,67 |
| 14 | Male care ~ prec.ann+prec.st.ann | 36,32 | 3,19 |
| 15 | Male care ~ tmean.ann+tmn.st.ann+prec.st.ann | 36,71 | 3,58 |
| 16 | Male care ~ tmean.ann+tvar.ann+prec.st.ann | 36,73 | 3,60 |
| 17 | Male care ~ tvar.ann+prec.ann | 36,89 | 3,76 |
| 18 | Male care ~ tmn.st.ann+prec.ann | 37,18 | 4,05 |
| 19 | Male care ~ tmean.ann+prec.ann | 37,27 | 4,14 |
| 20 | Male care ~ tmean.ann+tmn.st.ann+prec.ann+prec.st.ann | 37,48 | 4,35 |
| 21 | Male care ~ tvar.ann+prec.ann+prec.st.ann | 37,71 | 4,58 |
| 22 | Male care ~ tmean.ann+tvar.ann+tmn.st.ann+prec.st.ann | 37,77 | 4,64 |
| 23 | Male care ~ tmean.ann+prec.ann+prec.st.ann | 38,28 | 5,15 |
| 24 | Male care ~ tmean.ann+tvar.ann+prec.ann | 38,70 | 5,57 |
| 25 | Male care ~ tvar.ann+tmn.st.ann+prec.ann | 38,78 | 5,65 |
| 26 | Male care ~ tmean.ann+tvar.ann+tmn.st.ann | 38,86 | 5,73 |
| 27 | Male care ~ tmean.ann+tmn.st.ann+prec.ann | 39,21 | 6,08 |
| 28 | Male care ~ tvar.ann+tmn.st.ann+prec.ann+prec.st.ann | 39,44 | 6,31 |
| 29 | Male care ~ tmean.ann+tvar.ann+prec.ann+prec.st.ann | 39,72 | 6,59 |
| 30 | Male care ~ tmean.ann+tvar.ann+tmn.st.ann+prec.ann | 40,69 | 7,56 |
| 31 | Male care ~ tmean.ann+tvar.ann+tmn.st.ann+prec.ann+prec.st.ann | 41,34 | 8,21 |

**Supplementary Table S5.** Climatic models on female care in salamanders ranked by AICc values. Supported models (within *Δi* < 2 from the best performing model) are highlighted with **bold** fonts. Abbreviations for climatic variables: *tmean.ann*: annual mean temperature; *tvar.ann*: within-year variance of monthly temperature means; *tmn.st.ann*: between-year stochasticity of annual mean temperatures; *prec.ann*: annual precipitations sums; *prec.st.ann*: between-year stochasticity of annual precipitation sums; *terrestrial_eggs*: the presence of terrestrial egg-laying.

| *Rank* | *Model formula* | *AICc* | *Δi* |
| --- | --- | --- | --- |
| **1** | **Female_attendance_binary ~ tmean.ann+terrestrial_eggs** | **56.88** | **0.00** |
| **2** | **Female_attendance_binary ~ tmean.ann+tvar.ann+terrestrial_eggs** | **58.29** | **1.41** |
| **3** | **Female_attendance_binary ~ terrestrial_eggs** | **58.32** | **1.44** |
| 4 | Female_attendance_binary ~ tmean.ann+tmn.st.ann+terrestrial_eggs | 58.99 | 2.12 |
| 5 | Female_attendance_binary ~ tvar.ann+terrestrial_eggs | 59.34 | 2.46 |
| 6 | Female_attendance_binary ~ tmn.st.ann+terrestrial_eggs | 60.26 | 3.38 |
| 7 | Female_attendance_binary ~ tmean.ann+tvar.ann+tmn.st.ann+terrestrial_eggs | 61.16 | 4.28 |
| 8 | Female_attendance_binary ~ tmean.ann+tvar.ann+prec.st.ann+terrestrial_eggs | 61.60 | 4.72 |
| 9 | Female_attendance_binary ~ tvar.ann+tmn.st.ann+terrestrial_eggs | 61.90 | 5.03 |
| 10 | Female_attendance_binary ~ tmean.ann+prec.st.ann+terrestrial_eggs | 62.51 | 5.63 |
| 11 | Female_attendance_binary ~ tmn.st.ann+prec.st.ann+terrestrial_eggs | 64.29 | 7.41 |
| 12 | Female_attendance_binary ~ tvar.ann | 64.80 | 7.93 |
| 13 | Female_attendance_binary ~ prec.st.ann | 64.90 | 8.02 |
| 14 | Female_attendance_binary ~ tmn.st.ann | 65.44 | 8.56 |
| 15 | Female_attendance_binary ~ tvar.ann+prec.st.ann+terrestrial_eggs | 65.46 | 8.58 |
| 16 | Female_attendance_binary ~ prec.st.ann+terrestrial_eggs | 66.00 | 9.12 |
| 17 | Female_attendance_binary ~ tmean.ann | 66.12 | 9.24 |
| 18 | Female_attendance_binary ~ tmean.ann+tmn.st.ann | 66.61 | 9.73 |
| 19 | Female_attendance_binary ~ tvar.ann+tmn.st.ann | 66.69 | 9.81 |
| 20 | Female_attendance_binary ~ tmean.ann+tvar.ann | 66.78 | 9.90 |
| 21 | Female_attendance_binary ~ tmn.st.ann+prec.st.ann | 66.96 | 10.08 |
| 22 | Female_attendance_binary ~ tmean.ann+prec.ann+terrestrial_eggs | 67.70 | 10.82 |
| 23 | Female_attendance_binary ~ tmean.ann+prec.st.ann | 68.10 | 11.22 |
| 24 | Female_attendance_binary ~ tvar.ann+prec.st.ann | 68.65 | 11.77 |
| 25 | Female_attendance_binary ~ tmean.ann+tmn.st.ann+prec.st.ann+terrestrial_eggs | 69.25 | 12.37 |
| 26 | Female_attendance_binary ~ tmean.ann+tmn.st.ann+prec.st.ann | 69.36 | 12.48 |
| 27 | Female_attendance_binary ~ tmean.ann+prec.ann+prec.st.ann+terrestrial_eggs | 69.60 | 12.72 |
| 28 | Female_attendance_binary ~ tmean.ann+tvar.ann+prec.ann+terrestrial_eggs | 69.66 | 12.78 |
| 29 | Female_attendance_binary ~ tmean.ann+tmn.st.ann+prec.ann+terrestrial_eggs | 69.98 | 13.10 |
| 30 | Female_attendance_binary ~ tvar.ann+tmn.st.ann+prec.st.ann | 70.48 | 13.60 |
| 31 | Female_attendance_binary ~ tmean.ann+tmn.st.ann+prec.ann | 70.55 | 13.67 |
| 32 | Female_attendance_binary ~ tmean.ann+tvar.ann+tmn.st.ann+prec.st.ann+terrestrial_eggs | 70.76 | 13.88 |
| 33 | Female_attendance_binary ~ tmean.ann+tvar.ann+prec.ann+prec.st.ann+terrestrial_eggs | 71.50 | 14.62 |
| 34 | Female_attendance_binary ~ tmean.ann+tmn.st.ann+prec.ann+prec.st.ann+terrestrial_eggs | 71.65 | 14.77 |
| 35 | Female_attendance_binary ~ tmean.ann+tvar.ann+tmn.st.ann+prec.ann+terrestrial_eggs | 71.89 | 15.01 |
| 36 | Female_attendance_binary ~ tmean.ann+tvar.ann+tmn.st.ann | 71.93 | 15.05 |
| 37 | Female_attendance_binary ~ prec.ann+terrestrial_eggs | 72.01 | 15.14 |
| 38 | Female_attendance_binary ~ tvar.ann+tmn.st.ann+prec.st.ann+terrestrial_eggs | 72.15 | 15.27 |
| 39 | Female_attendance_binary ~ tmn.st.ann+prec.ann+terrestrial_eggs | 72.22 | 15.34 |
| 40 | Female_attendance_binary ~ prec.ann+prec.st.ann+terrestrial_eggs | 72.35 | 15.47 |
| 41 | Female_attendance_binary ~ tmean.ann+tmn.st.ann+prec.ann+prec.st.ann | 72.42 | 15.54 |
| 42 | Female_attendance_binary ~ tvar.ann+prec.ann+terrestrial_eggs | 73.27 | 16.39 |
| 43 | Female_attendance_binary ~ tmean.ann+tvar.ann+tmn.st.ann+prec.ann+prec.st.ann+terrestrial_eggs | 73.55 | 16.67 |
| 44 | Female_attendance_binary ~ tvar.ann+tmn.st.ann+prec.ann+terrestrial_eggs | 73.86 | 16.98 |
| 45 | Female_attendance_binary ~ tmn.st.ann+prec.ann+prec.st.ann+terrestrial_eggs | 73.86 | 16.98 |
| 46 | Female_attendance_binary ~ tvar.ann+prec.ann+prec.st.ann+terrestrial_eggs | 73.87 | 16.99 |
| 47 | Female_attendance_binary ~ tmean.ann+tvar.ann+tmn.st.ann+prec.st.ann | 75.51 | 18.63 |
| 48 | Female_attendance_binary ~ tvar.ann+tmn.st.ann+prec.ann+prec.st.ann+terrestrial_eggs | 75.53 | 18.65 |
| 49 | Female_attendance_binary ~ prec.ann | 75.93 | 19.05 |
| 50 | Female_attendance_binary ~ tvar.ann+tmn.st.ann+prec.ann | 76.30 | 19.43 |
| 51 | Female_attendance_binary ~ tmean.ann+prec.ann | 76.32 | 19.44 |
| 52 | Female_attendance_binary ~ tmean.ann+tvar.ann+prec.st.ann | 76.47 | 19.59 |
| 53 | Female_attendance_binary ~ tmn.st.ann+prec.ann | 77.20 | 20.32 |
| 54 | Female_attendance_binary ~ prec.ann+prec.st.ann | 77.81 | 20.93 |
| 55 | Female_attendance_binary ~ tmean.ann+prec.ann+prec.st.ann | 78.06 | 21.19 |
| 56 | Female_attendance_binary ~ tmn.st.ann+prec.ann+prec.st.ann | 79.18 | 22.30 |
| 57 | Female_attendance_binary ~ tmean.ann+tvar.ann+tmn.st.ann+prec.ann | 79.62 | 22.74 |
| 58 | Female_attendance_binary ~ tmean.ann+tvar.ann+tmn.st.ann+prec.ann+prec.st.ann | 79.76 | 22.88 |
| 59 | Female_attendance_binary ~ tvar.ann+prec.ann | 80.38 | 23.50 |
| 60 | Female_attendance_binary ~ tmean.ann+tvar.ann+prec.ann | 80.94 | 24.06 |
| 61 | Female_attendance_binary ~ tvar.ann+prec.ann+prec.st.ann | 82.39 | 25.51 |
| 62 | Female_attendance_binary ~ tmean.ann+tvar.ann+prec.ann+prec.st.ann | 82.83 | 25.95 |
| 63 | Female_attendance_binary ~ tvar.ann+tmn.st.ann+prec.ann+prec.st.ann | 84.55 | 27.67 |

References for Supplementary Data

(Vági et al.: The evolution of parental care in salamanders)

1. Akiyama S, Iwao Y, Miura I. 2011 Evidence for true fall–mating in Japanese newt *Cynops pyrrhogaster*. *Zool. Sci.* **28**, 758–763. (doi: 10.2108/zsj.28.758)
2. Alcher M. 1981 Reproduction en élevage de *Euproctus platycephalus*. *Amphibia-Reptilia* **2**, 97–105 (doi: 10.1163/156853881X00131)
3. Alfimov AV, Berman DI. 2010 Reproduction of the Siberian salamander, *Salamandrella keyserlingii* (Amphibia, Caudata, Hynobiidae), in water bodies on permafrost in Northeastern Asia. *Biology Bulletin*, **37**, 807–822
4. Anderson JD, Williamson GK. 1973 The breeding season of Ambystoma opacum in the northern and southern parts of its range. *J. Herpetol.* **7**, 320–321.
5. Ashton RE Jr, Braswell AL. 1979 Nests and larvae of the Neuse River waterdog, Necturus lewisi (Brimley) (Amphibia: Proteidae). *Brimleyana* **1**, 15–22.
6. Bille T. 1998 Eggs and hatchlings of the Mexican salamander *Pseudoeurycea cephalica* (Caudata: Plethodontidae). *Revista de Biología Tropical* **46**, 447–452.
7. Boscherini A, Romano A. 2011 Parental care in *Salamandrina perspicillata* (Amphibia, Salamandridae): egg defence against caddisfly larvae. *North-Western J. Zool.* **7**, 167–170.
8. Brandon RA, Altig RG. 1973 Eggs and small larvae of two species of *Rhyacosiredon*. *Herpetologica* **29**, 349–351.
9. Briggler JT, Puckette WL. 2003 Observations on reproductive biology and brooding behavior of the Ozark zigzag salamander, *Plethodon angusticlavius*. *The Southwestern Naturalist* **48**, 96–100.
10. Buckley D, Alcobendas M, García–París M. 2009 The evolution of viviparity in salamanders (Amphibia, Caudata): Organization, variation, and the hierarchical nature of the evolutionary process. pp. 145–154 in *Evolución y Adaptación. 150 años después del Origen de las Especies*, Dopazo HJ, Arcadi A, SESBE (eds.). Valencia: Obrapropia, S. L.
11. Canterbury RA, Pauley TK. 1994 Time and mating and egg deposition of West Virginia populations of the salamander Aneides aeneus. *J. Herpetol*. **28**, 431–434.
12. Charney ND, Castorino JJ, Dobro MJ, Steely SL. 2014 Embryo development inside female salamander (*Ambystoma jeffersonianum–laterale*) prior to egg laying. *PLoS ONE* **9**, e91919. (doi: 10.1371/journal.pone.0091919)
13. Chen C, Yang J,Wu Y, Fan Z, Lu W, Chen S, Lipeng Yu L. 2016 The breeding ecology of a critically endangered salamander, *Hynobius amjiensis* (Caudata: Hynobiidae), endemic to Eastern China. *Asian Herpetological Research* **7**, 53–58. (doi: 10.16373/j.cnki.ahr.150050)
14. Colleoni E, Denoel M, Padoa–Schioppa E, Scali S, Ficetola GF. 2014 Rensch's rule and sexual dimorphism in salamanders: patterns and potential processes. *J. Zool.* **293**, 143–151.
15. Crump ML. 1995 Parental care. In: *Amphibian biology, volume 2: Social behavior* (eds Heathvole H, Sullivan BK), pp. 518 – 567. Chipping Norton: Surrey Beatty & Sons.
16. Crump ML. 1996 Parental care among the Amphibia. *Adv. Stud. Behav*. **25**, 109 – 144. (doi: 10.1016/S0065-3454(08)60331-9)
17. Davenport JM, Summers K. 2010 Environmental influences on egg and clutch sizes in lentic–and lotic–breeding salamanders. *Phyllomedusa* **9**, 87–88.
18. De Lisle SP, Rowe L. 2013 Correlated evolution of allometry and sexual dimorphism across higher taxa. *Am Nat.* **182**, 630–639
19. Della Rocca F, Vignoli L, Bologna MA. 2005 The reproductive biology of *Salamandrina terdigitata* (Caudata, Salamandridae). *Herpetol. J.* **14**, 273–278.
20. Diaz–Paniagua C. 1989 Oviposition behavior of *Triturus marmoratus pygmaeus*. *J. Herpetol*. **23**, 159–163.
21. Duellman WE, Trueb L 1986 *Biology of amphibians*. New York: McGraw–Hill.
22. Gresens J. 2004 An introduction to the Mexican axolotl (*Ambystoma mexicanum*). *Lab Animal* **33**, 41–44.
23. Hanken J. 1979 Egg development time and clutch size in two neotropical salamanders. *Copeia* **4**, 741–744. (doi: 10.2307/1443885)
24. Harrison JR. 1967 Observations on the life history, ecology and distribution of *Desmognathus aeneus aeneus* Brown and Bishop. *Am. Midl. Nat*. **77**, 356–370.
25. Hasumi M. 1994 Reproductive behavior of the salamander *Hynobius nigrescens*: monopoly of egg sacs during scramble competition*. J. Herpetol.* **28**, 264–267. (doi: 10.2307/1564635)
26. Hasumi M. 2015 Social interactions during the aquatic breeding phase of the family Hynobiidae (Amphibia: Caudata). *Acta Ethologica* **18**, 243–253.
27. Houck LD. 1977a Reproductive biology of a Neotropical salamander, Bolitoglossa rostrata. *Copeia* **1977**, 70–82.
28. Houck, LD. 1977b. *Reproductive patterns in Neotropical salamanders*. University of California, Berkeley, CA: ProQuest Dissertation Publishing.
29. Jaeger RG. 1981 Dear enemy recognition and the costs of aggression between salamanders. *Am. Nat.* **117**, 962–974
30. Jockusch EL. 1997 Geographic variation and phenotypic plasticity of number of trunk vertebrae in slender salamanders, Batrachoseps (Caudata: Plethodontidae). *Evolution* **51**, 1966–1982.
31. Jockusch, EL, Mahoney MJ. 1997 Communal oviposition and lack of parental care in Batrachoseps nigriventris (Caudata: Plethodontidae) with a discussion of the evolution of breeding behavior in plethodontid salamanders. *Copeia* **1997**, 697–705.
32. Kakegawa M, Hasumi M. 2017 Effects of controlled water temperatures on oviposition in a lotic–breeding and externally fertilizing salamander (*Hynobius kimurae*). *River Research and Applications* **33**, 1036–1043. (doi: 10.1002/rra.3162)
33. Kern MM, Nassar AA, Guzy JC, Dorcas ME. 2013 Oviposition site selection by spotted salamanders (*Ambystoma maculatum*) in an isolated wetland. *J. Herpetol.* **47**, 445–449. (doi: 10.1670/11–179)
34. Khattak S, Murawala P, Andreas H, Kappert V, Shuez M, Sandoval–Guzmán T, Crawford K, Tanaka EM. 2014 Optimized axolotl (*Ambystoma mexicanum*) husbandry, breeding, transgenesis and tamoxifen–mediated recombination. *Nature Protocols* **9**, 529–540.
35. Krenz JD, Sever DM. 1995 Mating and oviposition in paedomorphic Ambystoma talpoideum precedes the arrival of terrestrial males. *Herpetologica* **51**, 387–393.
36. Kusano T. 1980 Breeding and egg survival of a population of a salamander, *Hynobius nebulosus tokyoensis* Tago. *Res. Popul. Ecol.* **21**, 181–196. (doi: 10.1007/BF02513620)
37. Kuzmin SL, Dasgupta R, Mirina EM. 1994 Ecology of the Himalayan newt (*Tylototriton verrucosus*) in Darjeeling Himalayas, India. *Russian J. Herpetol.* **1**, 69–76.
38. Lannoo M (ed.) 2005. Amphibian Declines: The Conservation Status of United States Species. University of California Press
39. Lunghi E, Manenti E, Manca S, Mulargia M, Pennanti R, Ficetola GF. 2014 Nesting of cave salamanders (*Hydromantes flavus* and *H. italicus*) in natural environments. *Salamandra* **50**, 105–109.
40. Lunghi E et al. 2018 Comparative reproductive biology of european cave salamanders (genus *Hydromantes*): nesting selection and multiple annual breeding. *Salamandra* **54**, 101–108.
41. Marvin GA. 1996 Life history and population characteristics of the salamander *Plethodon kentucki* with a review of *Plethodon* life histories. *Am. Midl. Nat.* **136**, 385–400. (doi: 10.2307/2426742)
42. McCranie JR, Wilson LD. 1992 *Nototriton barbouri* reproduction. *Herp. Rev.* **23**, 115–116.
43. Michaels CJ. 2016 Successful reproduction in *Paramesotriton chinensis* after more than a decade of reproductive inactivity, with observation of parental care. *Herpetological Bulletin* **137**, 24–27.
44. Milanovich JR, Trauth SE, Saugey DA, Jordan RR. 2006 Fecundity, reproductive ecology, and influence of precipitation on clutch size in the western slimy salamander (*Plethodon albagula*). *Herpetologica* **62**, 292–301.
45. Nussbaum RA. 1985 The evolution of parental care in salamanders. Ann Arbor, MI: The University of Michigan Press.
46. Nussbaum RA. 1987 Parental care and egg size in salamanders: An examination of the safe harbor hypothesis. *Res. Popul. Ecol.* **29**, 27–44. (doi: 10.1007/BF02515423)
47. Nussbaum, RA. 2003 Parental care. In *Reproductive Biology and Phylogeny of Urodela* (DM Sever, ed). Enfield, NH: Science Publishers,.
48. Ohdachi S. 1994 Growth, metamorphosis, and gape–limited cannibalism and predation on tadpoles in larvae of salamanders *Hynobius retardatus*. Zoological Science **11**, 127–131.
49. Oneto F, Ottonello D, Pastorino MV, Salvidio S. 2010 Posthatching parental care in salamanders revealed by infrared video surveillance. *J. Herpetol.* **44**, 649–653. (doi: 10.1670/09–181.1)
50. Park D. 2005 The first observation of breeding of the long–tailed clawed salamander, *Onychodactylus fischeri*, in the field. *Curr. Herpetol.* **24**, 7–12. (doi: 10.3105/1345–5834)
51. Phimmachak S, Stuart BL, Sivongxay N. 2012 Distribution, natural history, and conservation of the Lao newt (*Laotriton laoensis*) (Caudata: Salamandridae) *J. Herpetol*. **46**, 120–128. (doi: 10.1670/11–044)
52. Reinhard S, Voitel S, Kupfer A. 2013 External fertilisation and paternal care in the paedomorphic salamander *Siren intermedia* Barnes, 1826. *Zoologischer Anzeiger* **253**, 1–5. (doi: 10.1016/j.jcz.2013.06.002)
53. Ringia AM, Lips KR. 2007 Oviposition, early development and growth of the cave salamander, *Eurycea lucifuga*: surface and subterranean influences on a troglophilic species. *Herpetologica* **63**, 258–268. (doi: 10.1655/0018–0831)
54. Rollinson N, Rowe L. 2018 Oxygen limitation at the larval stage and the evolution of maternal investment per offspring in aquatic environments. *Am. Nat.* **191**, 604–619.
55. Russell KR, Mabee TJ, Cole MB. 2004 Distribution and habitat of Columbia torrent salamanders at multiple scales in managed forests of Northwestern Oregon. *J. Wildl. Manage.* **68**, 405–417
56. Sever DM. 1991 Sperm storage and degradation in the spermathecae of the salamander *Eurycea cirrigera. J. Morph.* **207**, 283–301
57. Sparreboom M, Ota H. 1995 Notes on the life history and reproductive behaviour of *Cynops ensicauda popei* (Amphibia: Salamandridae*). J. Herpetol.* **5**, 310–315.
58. Sparreboom M. 1983 On the sexual behaviour of *Paramesotriton caudopunctatus* (Liu & Hu) (Amphibia: Caudata: Salamandridae). *Amphibia-Reptila* **4**, 25–33.
59. Takahashi MK, Okada S, Fukuda Y. 2017 From embryos to larvae: seven–month–long paternal care by male Japanese giant salamander. *J. Zool.* 302, 24–31. (doi: 10.1111/jzo.12433)
60. Tanner WW. 1999 Additional comments on the nesting behavior of *Batrachoseps wrighti* (Bishop). *Great Basin Naturalist* **59**, 387–389.
61. Thompson EL, Gates JE, Taylor GJ. 1980 Distribution and breeding habitat selection of the Jefferson salamander, Ambystoma jeffersonianum, in Maryland*. J. Herpetol.* **14**, 113–120.
62. Trochet A, Moulherat S, Calvez O, Stevens VM, Clobert J, Schmeller DS. 2014 A database of life–history traits of European amphibians *Biodivers. Data J*. **2**, e4123. (doi: 10.3897/BDJ.2.e4123)
63. Vanwormer, E. 2000. "*Eurycea bislineata*" (On–line), Animal Diversity Web. Accessed October 03, 2020 at https://animaldiversity.org/accounts/Eurycea_bislineata/
64. Wells KD. 2007 *The ecology and behaviour of amphibians*. Chicago, IL and London, UK: Univ. of Chicago Press.
65. Williams SR. 1978 Comparative reproduction of the endemic New Mexico plethodontid salamanders, Plethodon neomexicanus and Aneides hardii. *J. Herpetol.* **12**, 471–476.
66. Xie F, Fei L, Ye C, Cai C, Wang ZW, Sparreboom M. 2000 Breeding migration and oviposition of the Chinhai salamander, *Echinotriton chinhaiensis*. *J. Herpetol.* **10**, 111–118.
67. Xiong J, Liu X, Zhang X, Li M. 2016 Sexual dimorphism in the yellow–spotted salamander, *Pseudohynobius flavomaculatus* (Urodela: Hynobiidae). *Russian J. Herpetol.* **23**, 235–238.
68. Zhang X, Xiong JL, Lv YY, Zhang L & Sun YY. 2014 Sexual size and shape dimorphism in the Wushan salamander, *Liua shihi* (Liu, 1950) (Urodela: Hynobiidae). *Italian J. Zool.* **81**, 368–373. (doi: 10.1080/11250003.2014.920927)
